# Application of machine learning algorithm in predicting distant metastasis of T1 gastric cancer

**DOI:** 10.1038/s41598-023-31880-6

**Published:** 2023-04-07

**Authors:** HuaKai Tian, Zitao Liu, Jiang Liu, Zhen Zong, YanMei Chen, Zuo Zhang, Hui Li

**Affiliations:** 1 Department of General Surgery, First Affiliated Hospital of Nanchang University, Nanchang, China; 2 Department of Gastrointestinal Surgery, Second Affiliated Hospital of Nanchang University, Nanchang, China; 3 Department of Obstetrics and Gynecology, Second Affiliated Hospital of Nanchang University, 1 MinDe Road, Nanchang, 330006 China; 4 Department of Rheumatology and Immunology, First Affiliated Hospital of Nanchang University, Nanchang, 330006 China

**Keywords:** Cancer, Gastroenterology, Risk factors

## Abstract

Distant metastasis (DM) is relatively uncommon in T1 stage gastric cancer (GC). The aim of this study was to develop and validate a predictive model for DM in stage T1 GC using machine learning (ML) algorithms. Patients with stage T1 GC from 2010 to 2017 were screened from the public Surveillance, Epidemiology and End Results (SEER) database. Meanwhile, we collected patients with stage T1 GC admitted to the Department of Gastrointestinal Surgery of the Second Affiliated Hospital of Nanchang University from 2015 to 2017. We applied seven ML algorithms: logistic regression, random forest (RF), LASSO, support vector machine, k-Nearest Neighbor, Naive Bayesian Model, Artificial Neural Network. Finally, a RF model for DM of T1 GC was developed. The AUC, sensitivity, specificity, F1-score and accuracy were used to evaluate and compare the predictive performance of the RF model with other models. Finally, we performed a prognostic analysis of patients who developed distant metastases. Independent risk factors for prognosis were analysed by univariate and multifactorial regression. K-M curves were used to express differences in survival prognosis for each variable and subvariable. A total of 2698 cases were included in the SEER dataset, 314 with DM, and 107 hospital patients were included, 14 with DM. Age, T-stage, N-stage, tumour size, grade and tumour location were independent risk factors for the development of DM in stage T1 GC. A combined analysis of seven ML algorithms in the training and test sets found that the RF prediction model had the best prediction performance (AUC: 0.941, Accuracy: 0.917, Recall: 0.841, Specificity: 0.927, F1-score: 0.877). The external validation set ROCAUC was 0.750. Meanwhile, survival prognostic analysis showed that surgery (HR = 3.620, 95% CI 2.164–6.065) and adjuvant chemotherapy (HR = 2.637, 95% CI 2.067–3.365) were independent risk factors for survival prognosis in patients with DM from stage T1 GC. Age, T-stage, N-stage, tumour size, grade and tumour location were independent risk factors for the development of DM in stage T1 GC. ML algorithms had shown that RF prediction models had the best predictive efficacy to accurately screen at-risk populations for further clinical screening for metastases. At the same time, aggressive surgery and adjuvant chemotherapy can improve the survival rate of patients with DM.

## Introduction

Gastric cancer (GC), ranking fifth in morbidity and third in mortality, is one of the most common malignant tumors of the digestive system^1,2^. As is well known, it is a relatively long process for tumors to occur and progress and significant differences exist for the clinical manifestations and prognosis among each stage ^3^. Early gastric cancer (EGC) means that the tumor has not invaded the submucosa, which is defined as T1 stage, regardless of lymph node metastasis^4^. Patients with EGC can obtain a better prognosis after radical surgical resection, while distant organ metastasis regarded as advanced stage represents poor prognosis^5^. By means of local invasion, hematogenous and lymphatic metastasis, the metastasis of tumors exists throughout the whole process, which implies that distant metastasis(DM) might also occur in the T1 stage ^6^.

Studies have indicated that the occurrence and development of stage IV GC is related to many factors, among which T-stage is an independent risk factor for DM^7^. As tumors invade deeper, the possibility of metastasis increases significantly. Since this depth of T1 is superficial and the tumor is only located in the mucous membrane or submucosa, most scholars hold that there is little probability of distant metastasis^8^. However, it is precisely this traditional cognitive point of view that leads to deficiencies or neglect in the preoperative diagnosis of T1 GC, delaying the optimal time for treatment and affecting the prognosis of patients^9^. At present, the preoperative examination of GC mostly depends on imaging methods such as CT, but the accuracy of imaging examination in the detection of DM is obviously insufficient^10^. However, it is undeniable that accurate preoperative diagnosis and prediction of DM in GC patients are crucial for guiding clinical treatment and improving the prognosis of patients.

In recent years, the treatment of stage IV GC has long been controversial^11^. In line with the treatment guidelines of the Japan Gastric Cancer Association, the treatment of stage IV GC mainly includes radiotherapy, chemotherapy, optimal supportive treatment and palliative surgery ^12^. It has been reported that the prognosis of stage IV GC is affected by many factors, among which the method of treatment is an independent risk factor for prognosis, as well as T-stage^7^. Radical resection or endoscopic resection performs well in the therapies for early gastric cancer and usually brings a better prognosis. Once DM occurs in T1 GC, however, the options of treatment and prognosis would be much different^13,14^. Therefore, this study constructed a predictive model for DM in T1 GC, screened the best predictive model by a machine learning(ML) algorithm, and further analysed the prognosis of patients with T1NxM1 gastric cancer, to better guide clinical diagnosis and treatment.

## Materials and methods

### Patients and samples

**Figure 1:**
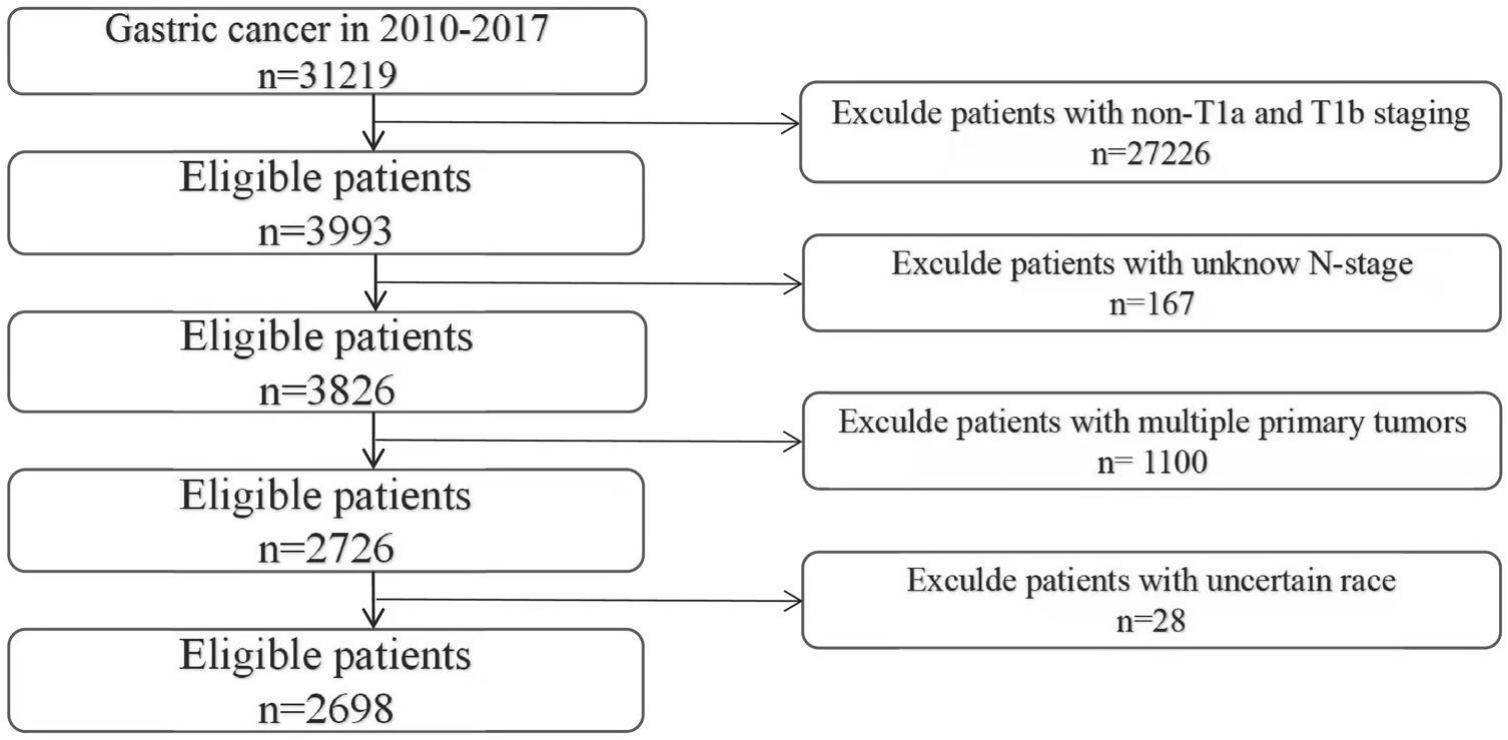
Flow chart of data screening.

Patients from the Surveillance, Epidemiology, and End Results (SEER) database (https://seer.cancer.gov/data/) were retrieved and downloaded via SEER * stat version 8.3.9 Software by the account 15962-Nov2020. Detailed data include information from 2010 to 2017, as specific T1 staging information is only available in 2010 and later. Meanwhile, we collected clinical data of patients with stage T1 gastric cancer admitted to the Second Affiliated Hospital of Nanchang University from January 2015 to January 2017. The study passed the hospital's ethical review (The Examination and Approval NO.Review [2018]No,(104)).Inclusion criteria: (1) Diagnosed as stage T1 gastric cancer (T1aNxMx and T1bNxMx); (2) Complete survival information.; (3) No pre-operative radiotherapy or immunotherapy. Exclusion criteria: (1) Suffering from multiple in situ tumors; (2) Tumor staging is incomplete; (3) The information is incomplete. Tumor diagnosis based on primary tumor site, grade and histology is coded in *International* *Classification* *of* Diseases for *Oncology*, *Third* *Edition* (ICD-O-3), and the seventh edition of AJCC staging system (the 7th AJCC edition) was applied for tumor-node-metastasis (TNM) stage system. The patient screening process was shown in Fig. 1.

### Data and variables selection

In the research, we considered 11 variables totally which was divided into three categories. Population characteristic variables include sex (Male, Female), age (< 40, 40–60, 60–80, > 80). Clinicopathological variables include tumor size (< 2 cm, 2-5 cm, > 5 cm, NA),tumor location (Fundus, Body, Antrum, Pylorus, Lesser curve, Greater curve, Overlapping, NOS), grade (Well, Moderate, Poorly, Undifferentiated, NA), M-stage (M0, M1), N-stage (N0, N1, N2, N3) and T-stage (T1a, T1b). Treatment variables include surgery, chemotherapy and radiotherapy.

### Statistical methods

**Figure 2:**
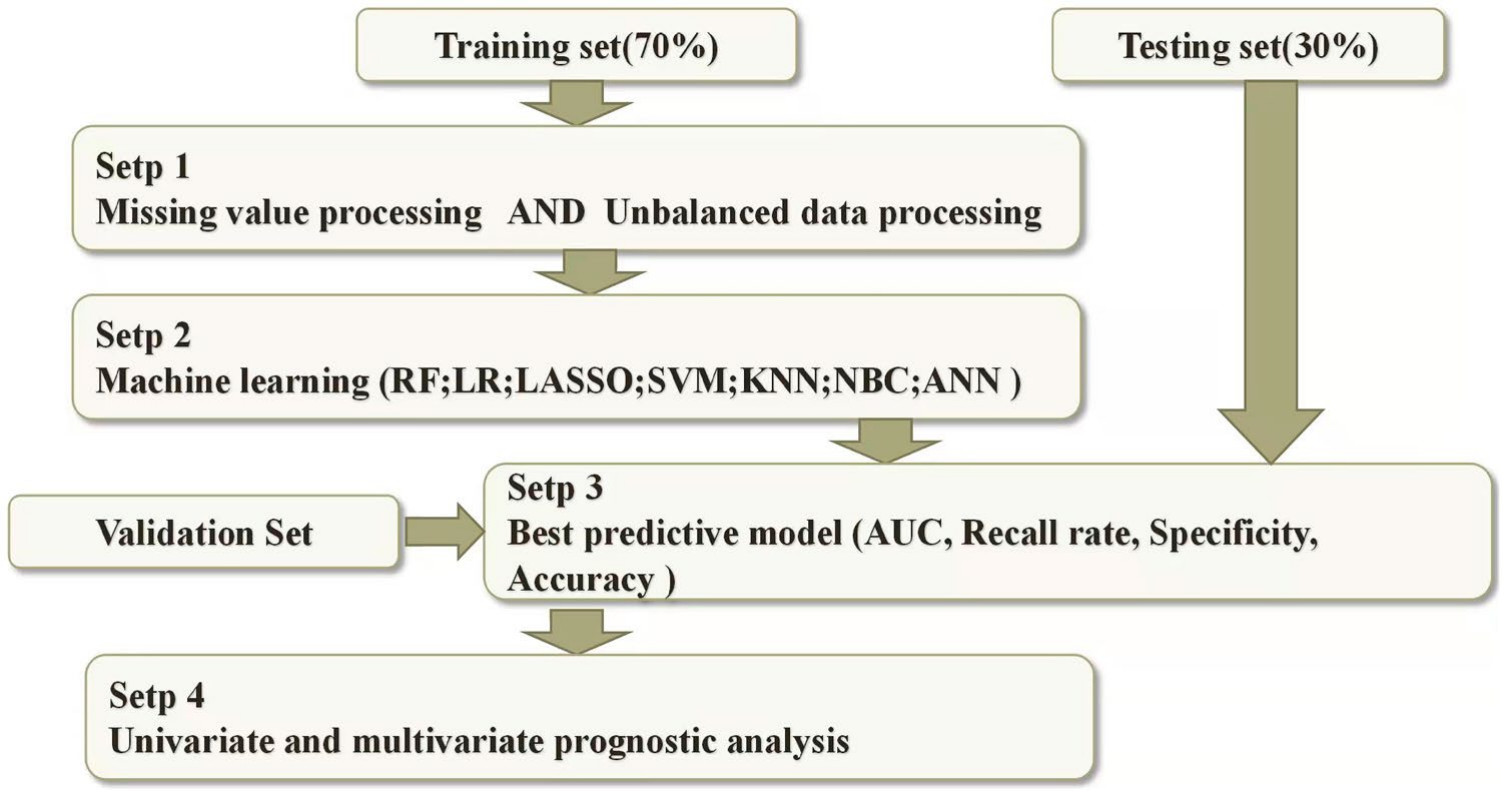
Flow chart of statistical analysis.

All statistical analyses were performed by R4.1.0 software and SPSS 24.0. The flow of this study was shown in Fig. 2. Heat maps were drawn for correlation analysis between variables including sex, age, tumour size, grade, T-stage, N-stage and tumour location. Independent risks affecting distant metastases from stage T1 gastric cancer were screened by logistic regression analysis. The results are represented by hazard ratios (HRs) and 95% confidence intervals (CIs). All patients were randomly divided 7:3 into a training set and a test set, and hospital patients were used as the external verification set. The training set developed the predictive model and the test set was evaluated for validation. We built seven ML algorithms in the training set: Logistic Regression (LR), Random Forest (RF), LASSO, Support Vector Machine (SVM), k-Nearest Neighbor (KNN), Naive Bayesian Model (NBC), and Artificial Neural Network (ANN). ROCAUC, sensitivity, specificity, F1-score and accuracy were used to compare the performance of the models. A test set further evaluated the validation. The external validation set validated the best predictive model to assess the generalisation capability of the model. For survival prognostic analysis, prognostic independent risk factors were analysed by univariate and multifactorial regression. K-M curves were used to express differences in survival prognosis for each variable and subvariable. For descriptive statistics, the chi-square test or Fisher's exact probability method were used to compare categorical variables. *P* < 0.05 indicated statistical significance.

### Ethics approval and consent to participate

As the study was conducted using a public database, patient informed consent and ethical review were not required.

## Results

### Patient characteristics

A total of 2698 patients were included in the SEER database for this study, 314 (11.64%) with distant metastases and 2384 (88.36%) without distant metastases. The external validation set consisted of 107 patients, 14 (13.08%) with distant metastases and 93 (86.92%) without distant metastases. the SEER database was randomised 7:3 into training and test sets. There was no statistical difference in age, sex, T-stage, N-stage, M-stage, chemotherapy, radiotherapy, tumour size, surgery, differentiation and Primary site between the two groups (*P* > 0.05). Table 1 shows the general characteristics of the patients in the three groups.

**Table 1:** Comparison of the general features of the training and test sets.

Variable	Training set (%) N = 1889	Test set (%) N = 809	*P* value	Validation set N = 107
**Age(years)**			0.7953	
< 40	59 (3.12%)	25 (3.09%)		8
40–60	405 (21.44%)	178 (22.00%)		67
60–80	971 (51.41%)	426 (52.66%)		31
> 80	454 (24.03%)	180 (22.25%)		1
**Sex**			0.2379	
Male	1050 (55.58%)	429 (53.03%)		71
Female	839 (44.42%)	380 (46.97%)		36
**T stage**			0.3458	
T1a	1054 (55.80%)	468 (57.85%)		40
T1b	835 (44.20%)	341 (42.15%)		67
**N stage**			0.3622	
N0	1553 (82.21%)	671 (82.94%)		81
N1	231 (12.23%)	96 (11.87%)		21
N2	80 (4.24%)	26 (3.21%)		4
N3	25 (1.32%)	16 (1.98%)		1
**M stage**			1	
M0	1669 (88.35%)	715 (88.38%)		93
M1	220 (11.65%)	94 (11.62%)		14
**Tumor size(cm)**			0.6843	
< 2	653 (34.57%)	276 (34.12%)		37
02-May	589 (31.18%)	261 (32.26%)		64
> 5	132 (6.99%)	47 (5.81%)		6
NA	515 (27.26%)	225 (27.81%)		0
**Differentiation**			0.081	
Well	233 (12.33%)	80 (9.89%)		1
Moderate	523 (27.69%)	230 (28.43%)		37
Poorly	853 (45.16%)	356 (44.00%)		63
Undifferentiated	28 (1.48%)	21 (2.60%)		6
NA	252 (13.34%)	122 (15.08%)		0
**Primary site**			0.9185	
Fundus	92 (4.78%)	35 (4.33%)		12
Body	303 (16.04%)	130 (16.07%)		29
Antrum	713 (37.74%)	306 (37.82%)		37
Pylorus	63 (3.34%)	35 (4.33%)		4
Lesser curve	233 (12.33%)	101 (12.48%)		12
Greater curve	99 (5.24%)	46 (5.69%)		1
Overlapping	135 (7.15%)	52 (6.43%)		12
NOS	251 (13.29%)	104 (12.86%)		0

### Comparison and analysis of model variables

**Figure 3:**
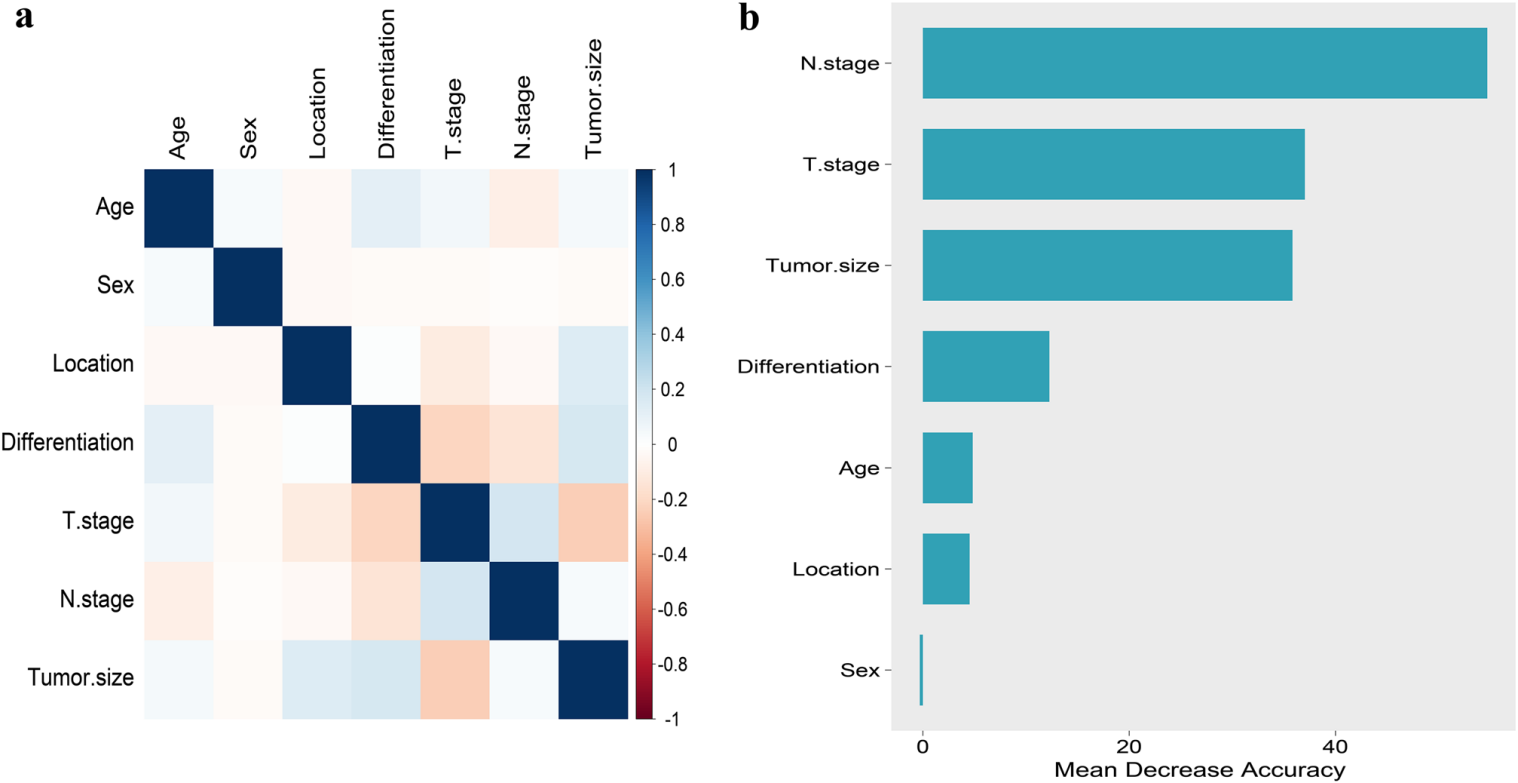
Results of Pearson correlation analysis for each variable and ranking of importance of predictive model characteristics.

First, we performed a Pearson correlation analysis between the variables (Fig. 3a). By stepwise backward LR analysis, we identified six characteristics as independent risk factors for predicting DM (Table 2), including age (*P* < 0.001), T-stage (*P* < 0.001), N-stage (*P* < 0.001), tumour size (*P* < 0.001), degree of differentiation (*P* = 0.002), and tumour site (*P* < 0.001). For the RF algorithm, the results of the analysis of variable significance showed (Fig. 3b) that N-stage, tumour size, T-stage, grade, age and tumour location were positively associated with distant metastases. Notably, this was consistent with the results of the multifactorial logistic regression model analysis.

**Table 2:** Multifactorial analysis of distant metastases from stage T1 gastric cancer.

Variable	Total (n = 2698) %	OR (95%CI)	*P* value
**Age(years)**			< 0.001
< 40	84 (3.11)	1 (Reference)	
40–60	583 (21.61)	1.301 (0.645–2.626)	
60–80	1397 (51.78)	0.913 (0.461–1.809)	
> 80	634 (23.50)	0.537 (0.259–1.111)	
**Sex**			0.21
Male	1479 (54.82)	1 (Reference)	
Female	1219 (45.18)	0.837 (0.634–1.106)	
**T stage**			< 0.001
T1a	1522 (56.41)	1 (Reference)	
T1b	1176 (43.59)	0.306 (0.218–0.430)	
**N stage**			< 0.001
N0	2224 (82.43)	1 (Reference)	
N1	327 (12.12)	5.539 (3.979–7.709)	
N2	106 (3.93)	1.941 (0.963–3.913)	
N3	41 (1.52)	1.972 (0.731–5.323)	
**Tumor size(cm)**			< 0.001
< 2	929 (34.43)	1 (Reference)	
2–5	850 (31.50)	4.068 (2.408–6.874)	
> 5	179 (6.63)	9.474 (5.057–17.748)	
NA	740 (27.43)	9.870 (5.977–16.297)	
**Differentiation**			0.002
Undifferentiated	49 (1.82)	1 (Reference)	
Poorly	1209 (44.81)	3.884 (0.873–17.275)	
Moderate	753 (27.91)	3.352 (0.744–15.109)	
Well	313 (11.60)	0.922 (0.176–4.844)	
NA	374 (13.86)	2.695 (0.593–12.251)	
**Primary site**			< 0.001
Fundus	127 (4.71)	1 (Reference)	
Body	433 (16.05)	0.449 (0.251–0.802)	
Antrum	1019 (37.77)	0.253 (0.146–0.439)	
Pylorus	98 (3.63)	0.356 (0.154–0.823)	
Lesser curve	334 (12.38)	0.269 (0.139–0.522)	
Greater curve	145 (5.37)	0.452 (0.211–0.968)	
Overlapping	187 (6.93)	0.688 (0.364–1.302)	
NOS	355 (13.16)	0.689 (0.395–1.204)	

### Establishment of a model for predicting distant Metastasis of T1 GC

**Figure 4:**
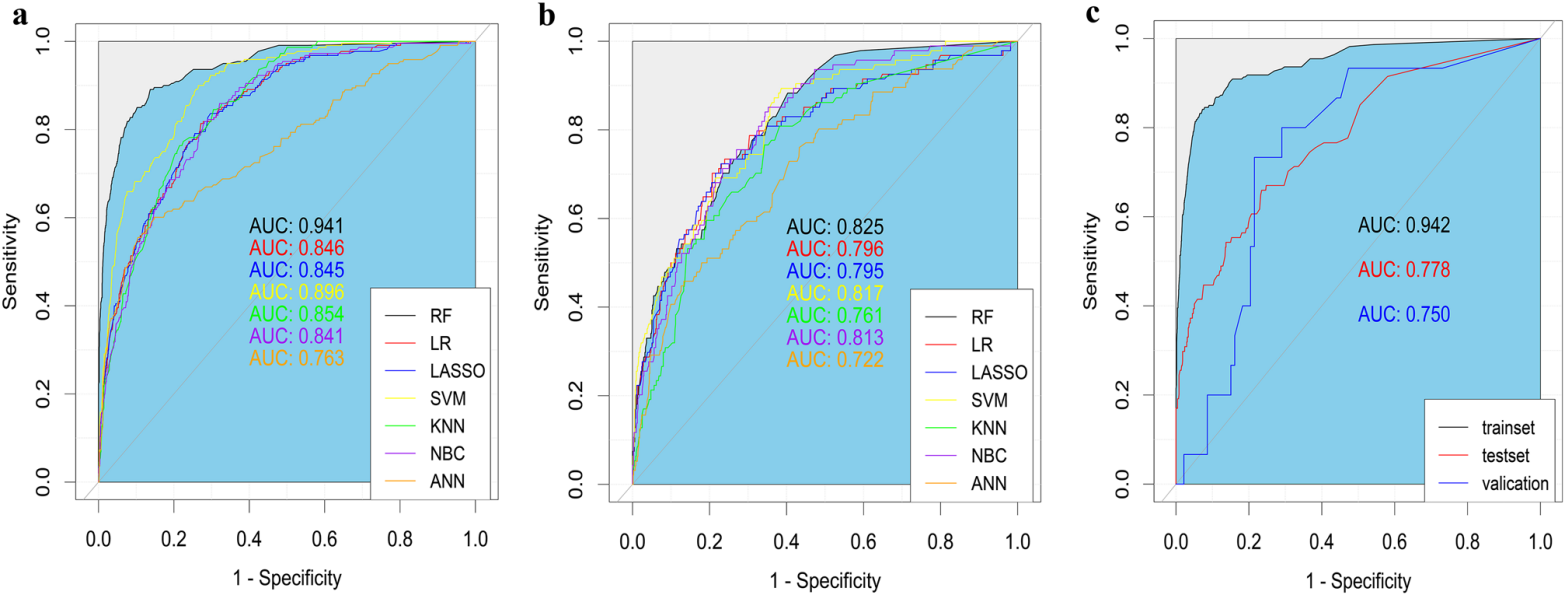
Receiver operating characteristic (ROC) curves for the training set, test set and external validation set prediction models [(**a**) training set; (**b**) test set; (**c**) external validation set].

We adjust the parameters of the training set to balance the model and avoid overfitting the model. Seven ML algorithms were performed on the balanced training set to construct the prediction model, and finally we found that the RF prediction model had the best prediction performance (AUC: 0.941, Accuracy: 0.917, Recall: 0.841, Specificity: 0.927, F1-score: 0.877) (Table 3, Fig. 4a). We further validated this in our test set and the results showed that the random forest prediction model had a ROCAUC of 0.825, which was significantly better than the other six models (Fig. 4b). Meanwhile, we validated the RF prediction model using 107 hospital patients as an external validation set (ROCAUC = 0.750) (Fig. 4c). Therefore, we believe that the RF prediction model can accurately predict the risk of developing DM in stage T1 GC.

**Table 3:** Comparison of predictive performance values of seven forecasting models in training set.

Models	AUC	Sensitivity (Recall)	Specificity	Accuracy	F1-score
RF	0.941	0.841	0.927	0.917	0.877
LR	0.846	0.773	0.797	0.795	0.784
LASSO	0.845	0.782	0.793	0.791	0.786
SVM	0.896	0.850	0.759	0.770	0.808
KNN	0.854	0.745	0.806	0.799	0.771
NBC	0.841	0.859	0.713	0.730	0.789
ANN	0.763	0.601	0.852	0.823	0.695

RF, random forest; LR, logistic regression; LASSO, least absolute shrinkage and selection operator; SVM, support vector machine; KNN, K-nearest neighbor; NBC, Naive Bayesian model; ANN, artificial neural network.

### Prognostic Analysis of patients with distant Metastasis of T1 GC

**Figure 5:**
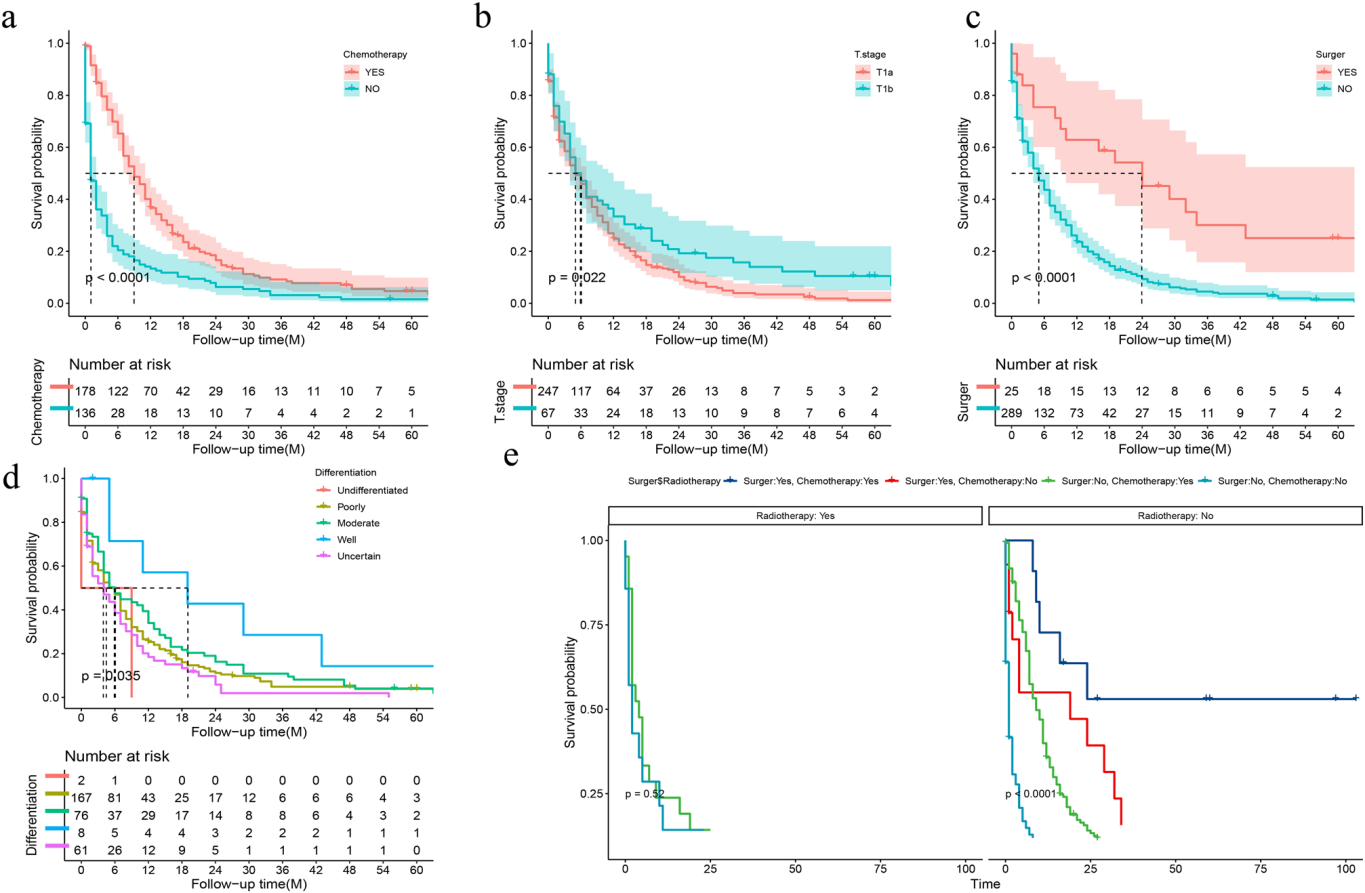
Survival prognosis analysis [(**a**) chemotherapy; (**b**) T-stage; (**c**) surgery; (**d**) differentiation; (**e**) surgery and adjuvant treatment].

To further analyze the prognosis of patients with distant metastasis in stage T1, we screened out the risk factors potential to influence the prognosis by univariate and multivariate regression analysis, and displayed the conclusion through K-M curve. Univariate analysis showed that chemotherapy (*P* < 0.001), surgery (*P* < 0.001), T stage (*P* < 0.022) and degree of differentiation (*P* < 0.035) were risk factors for prognosis (Fig. 5a–d). Multivariate regression analysis manifested that surgery and chemotherapy were independent risk factors for prognosis (Table 4). Additionally, subgroup analysis suggested that surgery combined with adjuvant chemotherapy could improve the survival rate of patients (Fig. 5e).

**Table 4:** Multifactorial analysis of survival prognosis of patients with distant metastases from stage T1 gastric cancer.

Variable	Total(n = 314) %	OR (95%CI)	*P* value
**T stage**			0.404
T1a	247 (78.66)	1 (Reference)	
T1b	67 (21.34)	0.881 (0.655–1.186)	
**Differentiation**			0.062
Undifferentiated	2 (0.64)	1 (Reference)	
Poorly	167 (53.18)	0.543 (0.134–2,201)	
Moderate	76 (24.20)	0.391 (0.095–1.608)	
Well	8 (2.55)	0.366 (0.073–1.847)	
NA	61 (19.43)	0.622 (0.151–2.554)	
**Surgery**			0.000
Yes	25 (7.96)	1 (Reference)	
NO	289 (92.04)	3.620 (2.164–6.065)	
**Chemotherapy**			0.000
Yes	178 (56.69)	1 (Reference)	
NO	136 (43.31)	2.637 (2.067–3.365)	

## Discussion

The prognosis of GC patients with distant metastasis is poor, with a 5-year survival rate < 5% and a median survival period of 11–18 months^5^. Extensive evidence has indicated that approximately 40% of patients have distant metastasis at the time of initial diagnosis of GC, and incidence increases as the tumor progresses^15,16^. Due to the high 5-year survival rate of T1 patients, many scholars have ignored the possibility of distant metastasis in T1 patients, especially in recent years when endoscopic treatment has gradually replaced traditional radical surgery^9,17^. A recent study showed that the probability of distant metastasis in patients with stage T1 is 8.17%^18^. Therefore, it is necessary to explore the risk factors and prognosis of distant metastasis of T1 gastric cancer. Meaningfully, this is the first study to construct a model for predicting distant metastasis of stage T1 gastric cancer through machine learning and analyse its survival and prognosis.

Previous studies have demonstrated that distant metastasis rarely occurs in T1 GC, which indicates a good prognosis for most patients with early gastric cancer^9^. Amazingly, in the present study, we found that the risk of distant metastasis in patients with T1 GC was as high as 11.64%. Thus, there is an urgent need to determine whether T1 patients have distant metastasis at the same time as the initial diagnosis. Conventional imaging tests (e.g., magnetic resonance imaging and computed tomography) can detect significant diffuse lesions, while positron emission tomography is a more reliable method of examining distant metastasis in GC especially in detecting micrometastases. However, it is limited by its effectiveness and practical costs^19^. Therefore, establishing a simple and effective prediction model can help clinicians identify high-risk patients for further examination and diagnosis.

Machine learning algorithms are a class of emerging methods that can accurately process raw data, analyse the relationships between important data, and make accurate decisions. One of the best features of machine learning algorithms is their excellent performance in predicting results in large databases, which is better than that of traditional regression methods^20^. In this study, we analysed and compared the prediction models established by seven ML algorithms, including logistic regression (LR), random forest (RF), LASSO, support vector machine (SVM), k-nearest neighbor (KNN), naive bayesian model (NBC), and artificial neural network (ANN). First, we used the training set to construct the prediction models and evaluated the efficacy values of the seven prediction models using AUC, sensitivity, specificity, F1-score and accuracy, and finally found that the RF model had the best prediction efficacy (AUC: 0.941, accuracy: 0.917, recall: 0.841, specificity: 0.927, F1-score: 0.877). The test set was used to further validate the results, which showed that the RF model was the optimal prediction model for predicting DM in stage T1 GC, with the best predictive efficacy (AUC = 0.825). The ability of the RF model to accurately predict DM in stage T1 gastric cancer was also confirmed by an external validation set (AUC = 0.750).The RF seems to be one of the most widely used and accurate machine learning models in clinical application research. Increasing evidence has reported that the random forest model is superior to other algorithms in dealing with data having a large number of features and highly nonlinear data, probably because the RF model uses more advanced classification decisions and different weight ratios compared to other models^21,22^. This study confirmed that the random forest prediction model can accurately predict the high-risk group with distant metastasis in T1 patients, which is conducive to further clinical examination for this population to develop better diagnosis and treatment strategies.

In this study,the 6 most important characteristics were included in the final RF prediction model: age, T-stage, N-stage, tumor size, grade and tumor site. The results suggested that the rate of DM in young patients (< 60 years old) is significantly higher than that in elderly patients (> 60 years old). Previous studies have reported that the rate of lymph node metastasis is higher in young GC patients^13,23,24^. More lymph node metastases in younger patients may be one of the reasons for distant metastases. Recently, accumulating studies have found that tumor biology plays a crucial role in the development of disease, which may be closely related to the occurrence and development of distant metastasis^25^. An additional study has shown that tumor size, depth of invasion and lymph node metastasis are significantly related to advanced gastric cancer^26^. Nevertheless, in our study we found that N stage and T stage were closely associated with distant metastasis. Interestingly, the rate of distant metastasis in patients with stage T1a was significantly higher than that in patients with stage T1b. This may result from that the lymph node metastasis occuring in submucosal patients first, while hematogenous metastasis occurs later in mucosal patients during infiltration into deeper layers. According to Japanese guidelines for the treatment of GC, patients with a tumor size > 2 cm have a significantly increased risk of metastasis and should receive radical resection for clean removal^12^. In addition, we found that the risk of distant metastasis increased significantly with tumor expansion, while this risk in patients with a tumor size > 5 cm was 8–9 times higher than that in patients with a tumor size < 2 cm. In our study, tumor site was one of the independent risk factors affecting distant metastasis in patients with T1 GC. Fundus tumors are prone to distant metastasis, which might be attributed to the wealth of blood vessels. Wealthy blood vessels are closely related to hematogenous metastasis. Moreover, our results showed that moderately and poorly differentiated GC patients are more likely to develop distant metastasis than undifferentiated differentiated and highly differentiated patients, which may be because cancer cells have invaded surrounding tissues, capillaries and lymphatic vessels, and these moderately and poorly differentiated tissues have a faster capacity of growth. This appears to be a departure from our previous understanding and requires further verification.

Subsequently, we also performed a prognostic survival analysis of patients with distant metastases. The results revealed that surgery (HR = 3.620, 95% CI 2.164–6.065) and adjuvant chemotherapy (HR = 2.637, 95% CI 2.067–3.365) were independent risk factors for survival and prognosis in patients with T1 distant metastasis. This is consistent with previous research^27^. Surgery for primary tumors may reduce the potential burden of immunosuppressive tumors and eliminate the source of further metastasis^28^. Hence, for patients with T1 distant metastases, aggressive surgery combined with adjuvant chemotherapy can greatly improve the prognosis of patients and improve the survival rate.

This study is the first to use an ML algorithm to predict DM in stage T1 GC, and it establishes an accurate predictive model to help identify people at high risk of DM at an early stage in the clinic. However, there are still some limitations in this study. First, as a retrospective study, the sample size of 2698 patients from 2010 to 2017 was relatively small. Next, the variables included in our study are finite, and other similar potential risk factors such as tumor markers, nutrition index and inflammation index are lacking, so a further model with more variables could improve the prediction accuracy.

In conclusion, we constructed and verified a prediction model of DM in patients with T1 GC through ML algorithm. The RF model has the best prediction efficiency and can accurately screen high-risk groups, providing help for further clinical metastasis screening. Meanwhile, our study also found that aggressive surgery and adjuvant chemotherapy can improve the survival rate of patients with DM.

## Supplementary Information

Supplementary Information 1.

Supplementary Information 2.

Supplementary Information 3.

Supplementary Information 4.

Supplementary Information 5.

## Data Availability

The datasets used and analyzed during the current study are available from the corresponding author on reasonable request.
