# Supplementary figures and images for: Application of machine learning algorithm in predicting distant metastasis of T1 gastric cancer
Source: Sci Rep. 2023 Apr 7;13:5741. doi: 10.1038/s41598-023-31880-6 (PMC10082185; doi:10.1038/s41598-023-31880-6)

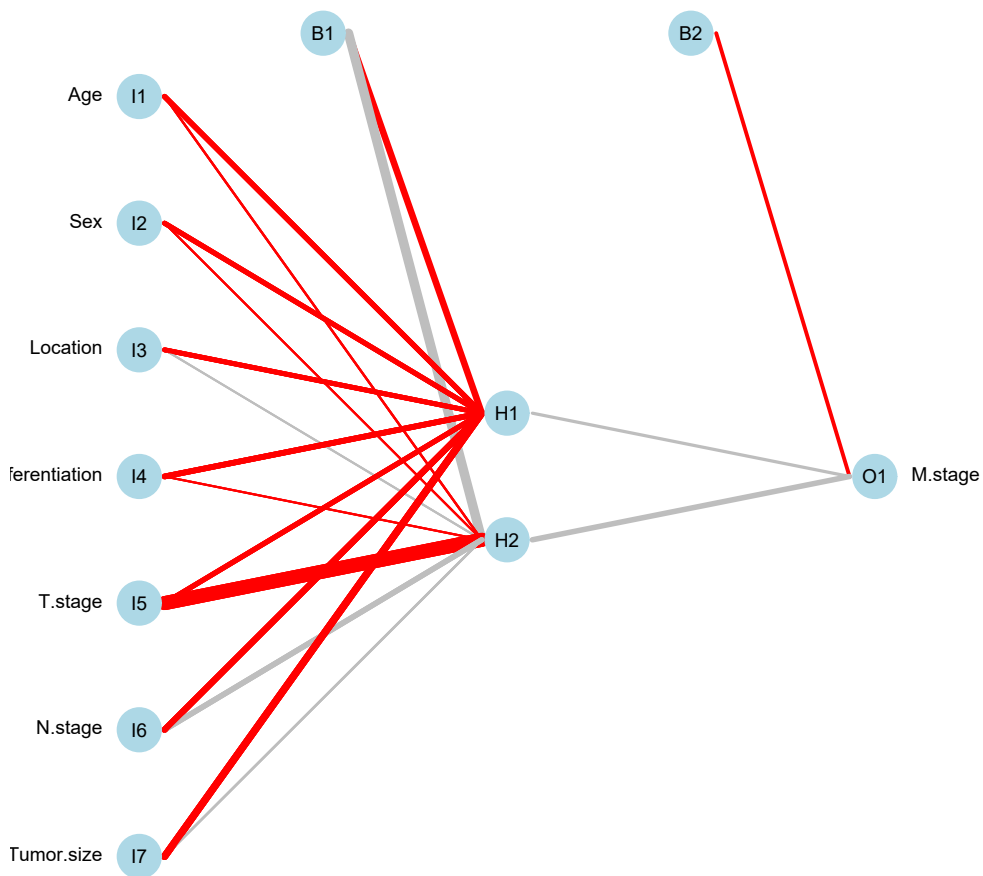

Supplement: Supplementary file 1 — Supplementary Information 1. [file 41598_2023_31880_MOESM1_ESM.pdf]

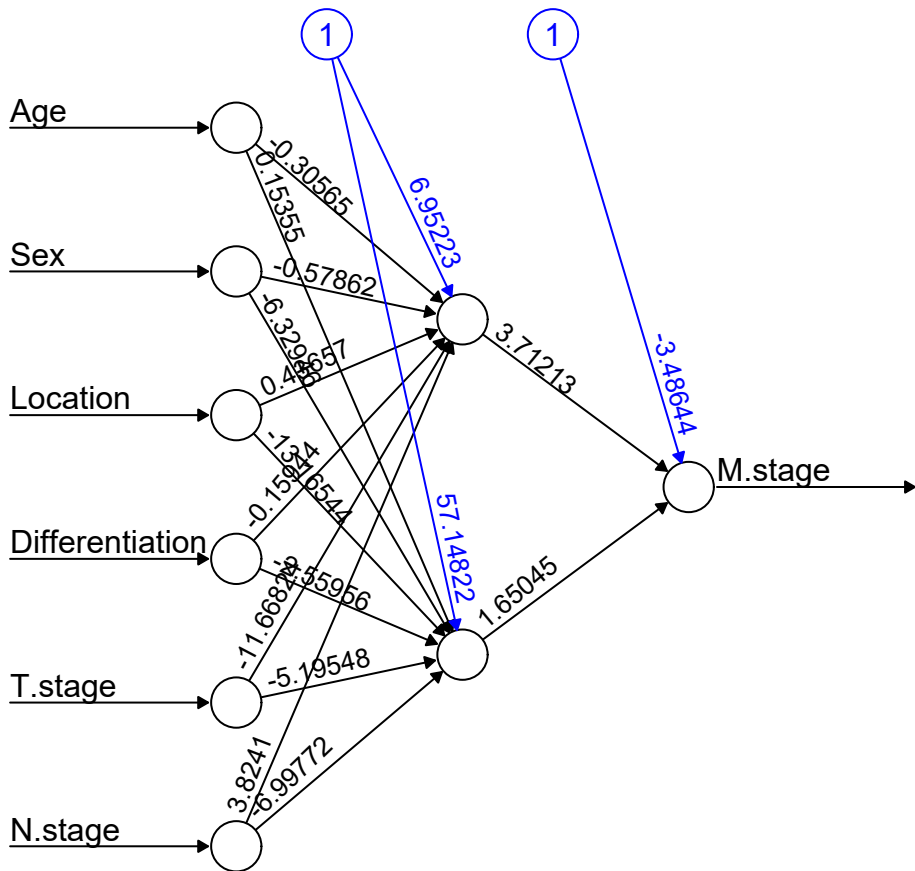

Error: 77.169685 Steps: 5085

Supplement: Supplementary file 2 — Supplementary Information 2. [file 41598_2023_31880_MOESM2_ESM.pdf]

Binomial Deviance

0.60  
0.65  
0.70  
0.75

7 7 6 6 6 4 3 2 1 0 0 0 0 0 0

-6 -5 -4 -3 -2 -1

$\text{Log}(\lambda)$

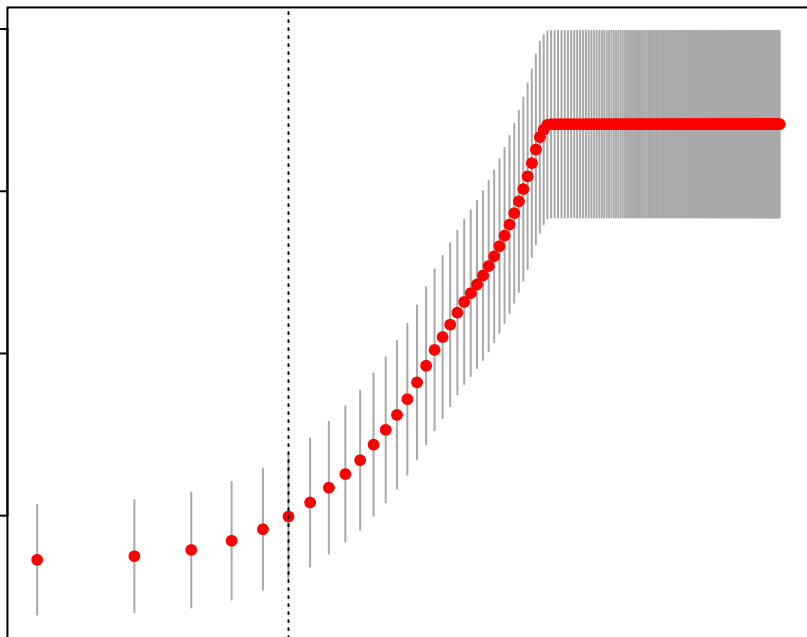

Supplement: Supplementary file 3 — Supplementary Information 3. [file 41598_2023_31880_MOESM3_ESM.pdf]

Coefficients

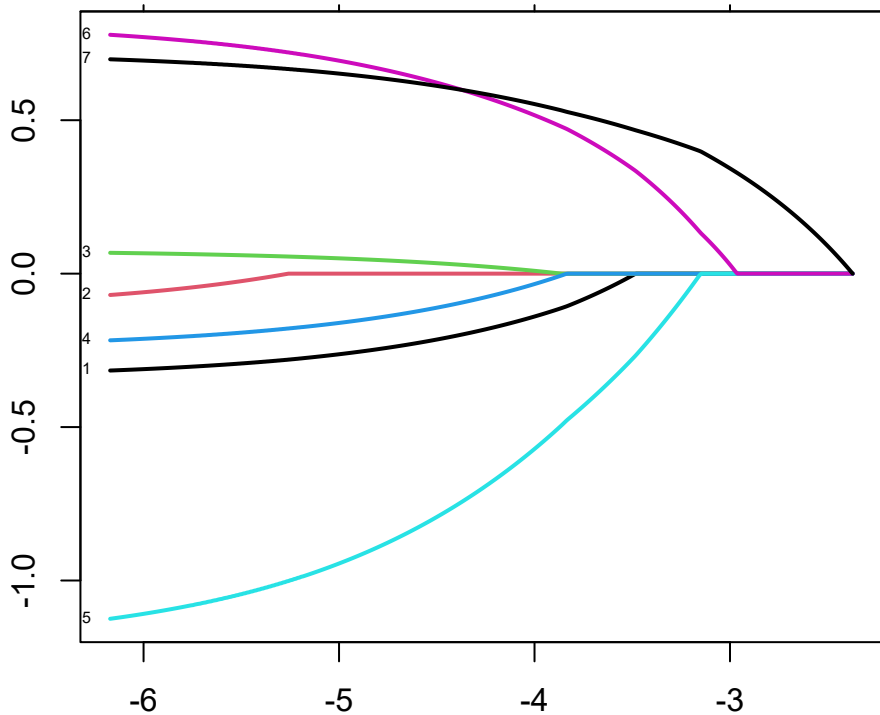

Log Lambda

Supplement: Supplementary file 4 — Supplementary Information 4. [file 41598_2023_31880_MOESM4_ESM.pdf]
